# Editorial: Food-derived plant polysaccharides: composition, bioavailability and health-promoting properties

**DOI:** 10.3389/fnut.2026.1984551

**Published:** 2026-09-28

**Authors:** Abul Hossain

**Affiliations:** Faculty of Land and Food Systems, The University of British Columbia, Vancouver, BC, Canada

**Keywords:** bioavailability, gut microbiota, health-promoting properties, plant polysaccharides, structure-activity relationship

Plant-derived polysaccharides are structurally diverse biomacromolecules increasingly recognized for their nutritional, functional, and health-promoting properties. Their biological activities are closely associated with structural characteristics including monosaccharide composition, molecular weight, glycosidic linkages, branching, conformation, and functional groups. Structural modifications can further influence these properties and their biological functions (1). Among these, sulfation has attracted particular interest because the degree and positional pattern of sulfate substitution can markedly influence polysaccharide bioactivity (2, 3). Despite substantial progress, establishing clear relationships among polysaccharide structure, gastrointestinal fate, bioavailability, and health-promoting effects remains a major challenge.

In launching the Research Topic “*Food-derived plant polysaccharides: composition, bioavailability and health-promoting properties*,” our aim was to advance understanding of plant-derived polysaccharides by connecting their structural and physicochemical characteristics with digestion, gut microbiota modulation, biological activities, and potential applications. This Research Topic brings together five contributions spanning structural characterization, simulated gastrointestinal digestion and fermentation, gut microbiota, immunomodulation, anticancer activity, plant-based dietary patterns, and the influence of harvesting and processing on plant bioactive constituents. Collectively, these studies illustrate how the health-promoting potential of plant polysaccharides and related bioactives depends on their composition and structure, stability during processing and digestion, and interactions with biological systems.

An important theme emerging from this Research Topic is the relationship between polysaccharide structure, gastrointestinal stability, and biological functionality. Li et al. investigated AABP-1B, a non-starch polysaccharide from *Anemarrhena asphodeloides* Bunge. AABP-1B was largely resistant to simulated upper gastrointestinal digestion and retained strong α-glucosidase inhibitory activity. During *in vitro* fermentation, it promoted commensal microorganisms, including *Bacteroides, Megasphaera*, and *Prevotella*, while suppressing potentially pathogenic bacteria and increasing short-chain fatty acid (SCFA) production. These findings connect digestive stability with microbial utilization and support the potential of AABP-1B as a prebiotic functional food ingredient. More broadly, plant-derived polysaccharides that escape upper gastrointestinal digestion can serve as substrates for intestinal microorganisms, with their structural characteristics influencing microbial utilization and prebiotic activity (4).

The gut microbiota also emerges as an important mediator of the immunomodulatory effects of plant polysaccharides. Yin et al. demonstrated that *Psoralea corylifolia* polysaccharides alleviated cyclophosphamide-induced immunosuppression in mice. These polysaccharides improved immune and antioxidant indicators and intestinal barrier integrity while altering gut microbial composition and metabolic profiles. Their effects were further associated with modulation of NF-κB and MAPK signaling and intestinal T-cell populations, supporting a microbiota-metabolite-immune signaling mechanism linking polysaccharide consumption with host immune function.

This mechanistic perspective aligns with the broader nutritional framework presented by Egas-Montenegro et al., who examined relationships between plant-based dietary patterns and the human gut microbiota. Their synthesis highlights dietary fiber and other plant-derived components as important modulators of microbial diversity and metabolism. Plant-rich dietary patterns may favor beneficial microbial profiles and increase metabolites such as SCFAs, with implications for inflammatory regulation, metabolic homeostasis, and intestinal barrier integrity. Together, these contributions reinforce the concept that the functionality of many plant polysaccharides extends beyond direct gastrointestinal interactions to include microbial transformation and the production of biologically active metabolites.

The Research Topic further highlights the importance of structure-activity relationships and direct cellular mechanisms. Wang et al. isolated and characterized SMP-3, an acidic polysaccharide from *Salvia miltiorrhiza* Bunge with pectin-like structural features and galacturonic acid as its predominant monosaccharide. SMP-3 selectively inhibited colorectal cancer cell proliferation and enhanced the anticancer effects of regorafenib. Combined treatment promoted cell-cycle arrest and apoptosis while suppressing migration and invasion through coordinated modulation of STAT3, AKT, ERK, and apoptosis-related signaling pathways. These findings illustrate how molecular and conformational characteristics may contribute to biological performance and underscore the importance of defining structure-activity relationships when developing polysaccharide-based functional ingredients. Structural modification offers an additional approach for tailoring physicochemical and biological properties (1), while sulfation provides a particularly relevant example in which the degree and position of substitution can influence biological activity (2, 3).

Importantly, realizing the functional potential of plant-derived compounds also requires consideration of raw material quality and post-harvest processing. Guo et al. demonstrated that harvesting age, harvest time, and drying method influenced the yield and bioactive composition of *Paeoniae Radix Rubra*. Under the regional conditions investigated, harvesting in late September of the fourth growing year followed by shade drying provided the best overall balance between yield and quality. Although this study evaluated a broader range of plant bioactive compounds rather than polysaccharides specifically, it emphasizes an important upstream consideration: cultivation, harvesting, and processing conditions can substantially influence the chemical and functional quality of plant-derived materials.

Taken together, the contributions to this Research Topic connect plant source and composition with structural characteristics, digestive stability, microbial fermentation, immune regulation, and disease-related cellular mechanisms. These findings also highlight opportunities for translating plant polysaccharides into functional foods, nutraceuticals, and delivery systems, although bioavailability remains an important determinant of their efficacy (5). Future research should therefore integrate advanced structural characterization with digestion models, microbiome profiling, metabolomics, and other omics approaches to better define structure-activity relationships and metabolic fate. Particular attention should be given to functional-group substitution and sulfation patterns, as well as the effects of processing on polysaccharide structure and functionality. Finally, much of the mechanistic evidence remains based on *in vitro* and animal models. Well-designed human studies are needed to establish bioavailability, efficacy, dose-response relationships, and long-term safety, ultimately supporting the development of evidence-based applications of food-derived plant polysaccharides.
